# Progression from Excessive to Deficient Syndromes in Chronic Hepatitis B: A Dynamical Network Analysis of miRNA Array Data

**DOI:** 10.1155/2013/945245

**Published:** 2013-04-16

**Authors:** Qi-Long Chen, Yi-Yu Lu, Gui-Biao Zhang, Ya-Nan Song, Qian-Mei Zhou, Hui Zhang, Wei Zhang, Shi-Bing Su

**Affiliations:** ^1^Research Center for TCM Complexity System, Shanghai University of TCM, Shanghai 201203, China; ^2^Shanghai Longhua Hospital, Shanghai University of TCM, Shanghai 200126, China

## Abstract

Traditional Chinese medicine (TCM) treatment is regarded as a safe and effective method for chronic hepatitis B (CHB), which requires a traditional diagnosis method to distinguish the TCM syndrome. In this study, we study the differences and similarities among excessive, excessive-deficient, and deficient syndromes, by an integrative and comparative analysis of weighted miRNA expression or miRNA-target network in CHB patients. We first calculated the differential expressed miRNAs based on random module *t*-test and classified three CHB TCM syndromes using SVM method. Then, miRNA target genes were obtained by validated database and predicted programs subsequently, the weighted miRNA-target networks were constructed for different TCM syndromes. Furthermore, prioritize target genes of networks of CHB TCM syndromes progression analyzed using DAVID online analysis. The results have shown that the difference between TCM syndromes is distinctly based on hierarchical cluster and network structure. GO and pathway analysis implicated that three CHB syndromes more likely have different molecular mechanisms, while the excessive-deficient and deficient syndromes are more dangerous than excessive syndrome in the process of tumorigenesis. This study suggested that miRNAs are important mediators for TCM syndromes classification as well as CHB development progression and therefore could be potential diagnosis and therapeutic molecular markers.

## 1. Introduction

The hepatitis B virus (HBV) is hepatotropic viruses that represent a serious global health problem [1]. The number of chronically infected subjects is estimated at 360 million for HBV, and annually these pathogens kill more than 1.5 million people worldwide [2]. Infection of hepatitis B virus (HBV) in human liver induces the development of chronic hepatitis B (CHB), liver cirrhosis, and in some instances hepatocellular carcinoma [3, 4]. Ever since the discovery of this virus, great efforts have been made towards the exploring of the molecular events and cellular signal transduction pathways that are altered by HBV infections, as well as the mechanisms that lead to the therapy of HBV infection diseases. 

In the past decades, substantial progress was made to treat CHB infections but no definitive cure is yet available. Although several antiviral drugs have been approved for CHB, however, this conventional treatment for HBV causes severe side effects and drug resistance [3]. Under these circumstances, many failing patients seek to get help from complementary and alternative medicine, especially, traditional Chinese medicine (TCM). Actually, many Chinese herbal medicines and their active compounds have been reported as a potential candidate for anti-HBV drugs [4].

TCM is a medical system, and discoveries of ancient Chinese evolved through at least 3000 years of uninterrupted clinical practice. The TCM treatment usually requires a traditional diagnosis method to distinguish the TCM syndrome, which is based on clinical symptoms and signs followed by the use of individualized treatment [5]. Interestingly, in the clinical outcomes, CHB patients with different TCM syndromes have responded differently to the same TCM therapy [6, 7]. This phenomenon suggests that TCM syndromes are significantly associated with disease. 

In the previous study, we reported the comparisons of microarray gene expression in HBV, the identification of significantly differentially expressed genes under the two major syndromes of CHB, including liver-gallbladder dampness heat syndrome (LGDHS) and liver depression and spleen deficiency syndrome (LDSDS) [8]. However, the molecular mechanism from excessive syndrome to deficient syndrome in CHB is still obscurity. 

MicroRNA (miRNA) is an endogenous, noncoding, single-stranded small RNA molecule of approximately 22 nucleotides and constitutes a novel class of gene regulators in animals and plants [9, 10], which inhibit translation or induce mRNA degradation in general by binding to the 3′-untranslated regions (3′-UTRs) of target mRNAs [11]. Generally, miRNAs are very stable in circulation systems, and tissue or organ. They are often detected in blood under pathological conditions, which is more likely caused by cell turnover, cell destruction, and pathological injury [12]. Systems biology approaches typically involve the identification of disease genes [13], dysfunctional pathways [14], network signatures, and drug-target networks [15] by high-throughput data analysis, which are expected to illuminate the interlinkage between genotypes and disease phenotypes and further reveal the biological mechanisms of complex diseases [16]. In this work, we hypothesized that miRNAs expression level is an important factor in CHB progression, and thus we focused on the analysis of the differences and similarities in the three syndromes of CHB, which comprised LGDHS, LDSDS, and (LKYDS) liver-kidney yin deficiency syndrome. The aim is to demonstrate the change process of molecular mechanism from excessive syndrome (LGDHS) to excessive-deficient syndrome (LDSDS), and then to deficient syndrome (LKYDS) at a network level by an integrative and comparative analysis of weighted miRNA-target network in CHB patients.

## 2. Materials and Methods

### 2.1. Clinical Specimens

Clinical serum collected from 9 CHB patients and 10 healthy donors (normals), who came from Shanghai Longhua Hospital in this work. Then, these serums were subjected to miRNA microarray analysis. The diagnostic criteria of western medicine for CHB followed the guidelines that were defined by the Chinese Society of Hepatology and Chinese Society of Infectious Diseases in 2005 [17]. The TCM syndrome system for CHB applied by the 3 seiner TCM doctors of each diagnosis was accepted according to the standards of TCM differential syndromes of viral hepatitis which were defined by the Internal Medicine Hepatopathy Committee of Chinese Traditional Medicine Association in 1991 [18]. This research project was approved with the local ethics committee of Shuanghai University of TCM, and all patients were informed consent for this study.

The differentiation between TCM syndromes in CHB patients were shown in Table 1. There were 3 LGDHSs, 3 LDSDSs, and 3 LKYDSs. In addition, 10 sera of control were random obtained from 120 individuals who had physical examination at Shanghai Longhua Hospital. 

### 2.2. Serum Sample Collection and RNA Isolation

All serum samples were from the peripheral venous blood of CHB patients and healthy donors, which were immediately frozen in liquid nitrogen and then stored at −80°C. The RNAs in serum were extracted using a miRVana PARIS kit (Ambion, Austin, TX, USA) according to the manufacturer's protocol, and based on the RNase-free DNase I (Promega, Madison, WI, USA) to eliminate DNA contamination. The concentration of RNAs isolated from serum ranged from 1.5 to 12 ng/*μ*L.

### 2.3. miRNA Microarray and Data Analysis

The profiles of serum miRNAs of 9 CHB patients and 10 controls were generated using Agilent Human miRNA microarray V3 (Agilent Technologies Inc., Santa Clara, CA, USA), 60 ng of RNA was labeled and hybridized for each array. Hybridization signals were detected with the Agilent microarray scanner; the data were extracted using Feature Extraction V10.7 (Agilent Technologies, CA, USA). All raw data were transformed to log2 and normalized each expression by having zero mean and unit sample variance.

In order to evaluate the dynamical progress form excessive syndrome to deficient syndrome in CHB, we compared the miRNAs expressions of LGDHS, LDSDS, and LKYDS to Normal, respectively. The relative miRNA expression levels were further normalized utilizing the median over all patients that make each patient have a median log ration of 0 in normalized expression levels. The weighted differences miRNAs between TCM syndromes were calculated using the random variance model *t*-test, in which the fold-change >1.5 and *P* < 0.05 were considered significant. Heatmap analysis and hierarchical cluster analysis of expression data were performed using Cluster 3.0 and TreeView programs. Class prediction was performed using a statistical algorithm of the support vector machine (SVM) incorporating miRNA differentially expressed at a unvariate parametric significance level of *P* = 0.01. The prediction rate was estimated via 10-fold and 10 times cross-validation and the bootstrap method for small sample data.

### 2.4. Identification and Prediction of miRNA Target Genes

Validated target genes selected to be done by miRNAs using TarBase 6.0, which hosts the largest collection of manually curate experimentally validated miRNA-gene interactions [19].

Unverified miRNA target genes were predicted to be regulated by miRNAs based on 10 programs, including DIANAmT, miRanda, miRDB, miRWalk, RNAhybrid, PicTar4, PicTar5, PITA, RNA22, and TargetScan. In these programs, the miRDB is different with others, which was using SVM learning machine to predict miRNA targets [20]. In order to increase the accuracy of predicted targets, we further screened prediction hits from two ways; that is, (i) random selected two sets data from 9 programs excepted miRDB, obtained the intersection of selected data, then united these intersection, and thus obtained Data A; (ii) selected the miRDB data whose the score >60, defined this data is Data B. Finally, the intersection between Data A and Data B acts as final data for build miRNA-target network. This strategy was more likely to yield higher target genes above background.

### 2.5. Enrichment Analysis of Target Genes

Of the inferred miRNA target genes, those showing a significant (*P* < 0.05) expression difference between Normal, LGDHS, LDSDS, and LKYDS samples were analyzed for pathways involving these genes using DAVID online analysis [21, 22], and significance analysis was determined when *P* values were corrected for false discovery rate (FDR). Gene sets containing less than 5 genes overlapping were removed from the DAVID analysis. In our analysis, GO terms and pathways with an FDR-adjusted *P*-value of less than 0.05 were retained.

### 2.6. Weighted miRNA-Target Network Construction

We built the weighted miRNA-target gene networks for different syndromes of CHB by computing the miRNA and target gene degree distribution based on experimental validated target genes and predicted target genes, thus inferring the miRNA-target network in 3 TCM syndromes of CHB, respectively. In the process of network building, miRNA nodes were weighted by their expression fold changes (absolute value of log2), while target genes were weighted based on degree distributions between consecutive groups and thus obtained a node-weighted miRNA-target interaction network for each stage. In order to validate the veracity of above network, rank all nodes (including miRNAs and target genes) of network according to their weights and tested the similarity of them [23], thereafter, obtained deregulated nodes for mapping the network of consecutive TCM syndromes progression. In the weighted miRNA-target network, the nodes represent miRNAs or genes, the edges represent the connection strength (adjacency), and the neighbors of each node >2 were selected in the network. 

## 3. Results and Discussion

### 3.1. Differential Expressed miRNAs between TCM Syndromes in CHB

**Figure 1:**
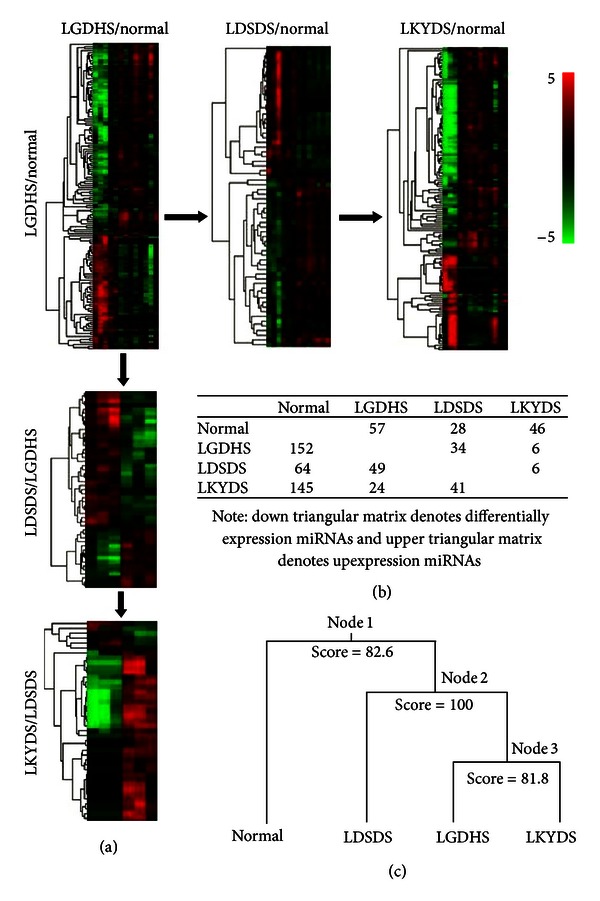
(a) Heatmap of differential expressed miRNAs among the LGDHS/normal, LDSDS/normal, and LKYDS/normal was shown at the horizontal, and the heatmap of differential expressed miRNAs between the LGDHS/LDSDS and LDSDS/LKYDS were shown at the vertical. (b) Differentially expressed miRNAs of three syndromes. (c) Relationship between four classes (normal and three syndromes) divided by binary tree classification.

The relative expressions of miRNAs were calculated and analyzed with random module *t*-test of R package, to search whether there are some significant differential expressed miRNAs among the consecutive stages form excessive syndromes to deficient syndromes. It was differentially expressed 157 miRNAs in LGDHS/Normal, 64 miRNAs in LDSDS/Normal, and 145 miRNAs in LKYDS/Normal. In the process from excessive to deficient syndromes, 49 differences in LGDHS/LDSDS and 41 miRNAs in LDSDS/LKYDS. Hierarchical cluster analysis revealed that the expression profiles of the differentially miRNAs from each syndrome were roughly classified, respectively. The consecutive heatmaps were shown in Figure 1(a), and differentially expressed miRNAs are shown in Figure 1(b). 

With the heatmaps, we noted that the miRNAs expression profiles of LGDHS/Normal and LKYDS/Normal were more closely. Thus, we attempted to differentiate miRNAs expression profiles into the samples using a supervised learning algorithm (binary tree classification) for classifying 3 TCM syndromes, and SVM acts as prediction method. As shown in Figure 1(c), the expression profiles were first classified into Normal and TCM syndromes (LGDHS, LDSDS, and LKYDS) in CHB (node 1) (score = 82.6). The LDSDS was then cluster to LGDHS and LKYDS (node 2) (score = 100), indicated that the Excessive-deficient combination syndrome (LDSDS) was distinctly difference for Excessive syndrome (LGDHS) and deficient syndrome (LKYDS). Interestingly, we note that the LGDHS clustered to LKYDS (node 3) (score = 81.8), they form a parallel branch and then cluster to LGDHS. Although the score between LGDHS and LKYDS is 81.8, which sufficiently supported the classification for them, however, this phenomenon still implicated the relationship between LGDHS and LKYDSs is more closely than LDSDS. 

For this strange event, we infer that the miRNA-regulated mechanism of LDSDS might have more complexity, because LDSDS is a complicated intergradation that form excessive syndrome to deficient syndrome, it has intersexuality features for two syndromes, synchronously. Whereas LGDHS and LKYDS are extrinsic more closely, they are purely single excessive syndrome and deficient syndrome, respectively. Acutely, the heatmaps also reveal that miRNAs expression levels are giant difference between LGDHS and LKYDS (Figure 1(a)). 

In overview of this study, the findings support the notion that the differential expressed miRNAs are distinctly associated with excessive (LGDHS), deficient syndrome (LKYDS), and Excessive-deficient combination syndromes (LDSDS). It means that the LGDHS, LDSDS, and LKYDS in CHB are completely different TCM syndrome based on miRNAs expression level.

### 3.2. Overview of the miRNA-Target Networks and Network Connections

As a class of gene regulators, combinatorial regulation is an important feature for miRNA. Usually, a given miRNA may have multiple different mRNA targets, whereas a given target gene may also be targeted by multiple miRNAs [24]. In this study, based on the expression ratios of miRNAs in each TCM syndrome, the target genes were obtained using the validation database (Tarbase 6.0) and 10 predicted programs. The number of predicted target genes was selected which actually showed significant differences (*P* < 0.05) in each prediction program. 

**Figure 2:**
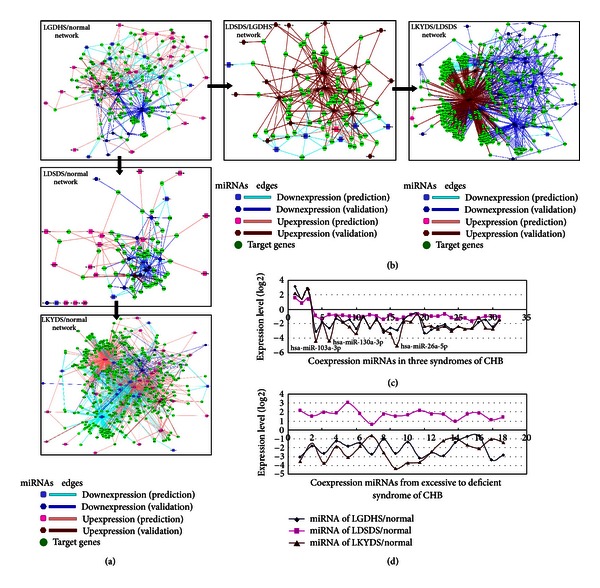
MiRNA-target networks of LGDHS, LDSDS, and LKYDS in CHB. (a) The networks that LGDHS, LDSDS, and LKYDS compare with normal, respectively. (b) The networks from excessive syndrome to excessive-deficient syndrome and deficient syndromes. (c) The miRNA expression levels in 3 TCM syndromes' networks. (d) The miRNA expression levels from excessive to deficient syndromes' networks.

Following the differential expressed miRNAs among LGDHS, LDSDS, and LKYDS in CHB, we built a miRNA-target network for each stage based on the validated and predicted target genes of miRNAs. The representative network was shown in Figure 2. These networks are highly weighted to evaluate the hub nodes (miRNAs or target genes) in terms of topological structure measurements. In the work, a hub node is defined to have more than 5 interactions in those stage-specific networks. In the different TCM syndromes, these hub nodes were highly significant to affect the network architecture; especially, it also implicates that the interesting potential modules were existent in the networks, which in biological networks often represent molecular complexes and pathways [23]. 

Although the successive networks of TCM syndromes in CHB were identified independently, they had relatives with respect to the nodes and interactions. As shown in Figure 2, the Excessive syndrome (LGDHS/Normal) network consists of 152 miRNAs, Excessive-deficient syndrome (LDSDS/Normal) network consists of 64 miRNAs, and Deficient syndromes (LKYDS/Normal) network consists of 145 miRNAs. Furthermore, have 38 coexpression miRNAs between LGDHS/Normal and LDSDS/Normal and 45 miRNAs in common between LDSDS/Normal and LKYDS/Normal, and LGDHS/Normal shares 82 miRNAs with LKYDS/Normal network. Notably, there were 31 co-expression miRNAs among the LGDHS/Normal, LDSDS/Normal, and LKYDS/Normal network, but only 3 miRNAs are upexpression. The 31 miRNAs expression levels of 3 TCM syndromes were shown in Figure 2(c). This poor overlap of up-expression miRNAs suggests a dramatic difference of deregulation in Excessive and Deficient syndromes and indicates that the LGDHS, LDSDS, and LKYDS are completely different syndrome from TCM classification in CHB.

Compare the process form Excessive syndrome to Deficient syndromes network, and the LGDHS/Normal network shares 48 miRNAs with LGDHS/LDSDS network (50 miRNAs), while the LGDHS/LDSDS network and LDSDS/LKYDS network (41 miRNAs) had 18 co-expression miRNAs. In addition, 18 miRNAs were co-expression among LGDHS/Normal, LGDHS/LDSDS, and LDSDS/LKYDS networks. Interestingly, we note that these 18 coexpression miRNAs are downexpression in LGDHS/Normal network and LDSDS/LKYDS network; however, they were all up-expression in LGDHS/LDSDS network (Figure 2(d)). In the classification of TCM syndromes in CHB, LGDHS is a typical Excessive syndrome, and LKYDS is a Deficient syndrome, whereas LDSDS more likely belongs to Excessive-deficient combination syndrome. The difference of miRNAs expressions among the 3 TCM syndrome networks indicated that the molecular mechanism has great varieties from Excessive to Deficient syndromes. 

### 3.3. Comparison of Network with CHB Syndromes Interacting miRNAs and Target Genes

**Figure 3:**
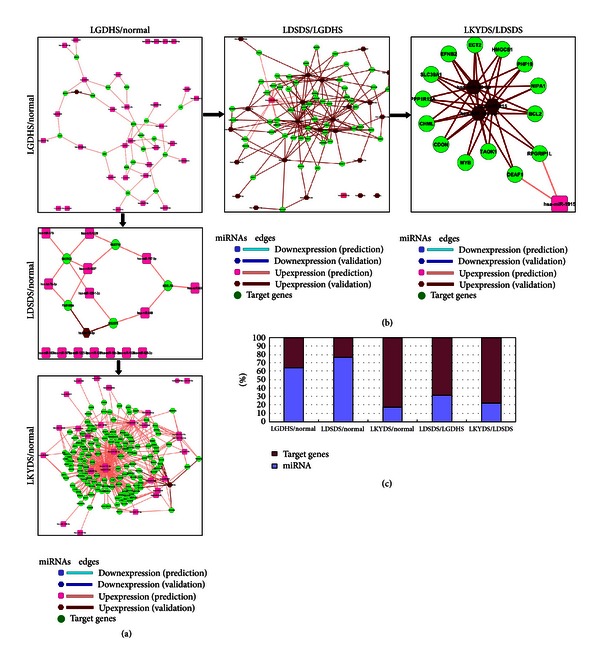
Upregulated miRNA-target networks of LGDHS, LDSDS, and LKYDS in CHB. (a) The networks that LGDHS, LDSDS, and LKYDS compare with normal, respectively. (b) The networks from excessive to deficient syndromes. (c) The percentage of miRNAs distribution and target genes in related TCM syndrome's network.

Because miRNA general inhibits translation or induces mRNA degradation by binding to the 3′-UTRs of target mRNAs [11], here, we attempted to construct the upregulated miRNA-target networks for each TCM syndrome. Figures 3(a) and 3(b) show the up-regulated network that the degree was not less than 5. All networks comprise CHB-related miRNAs and target genes, as was distributed in Figure 3(c). It is interesting to note that the proportion of hub miRNAs in LGDHS/Normal (64.0%) and LDSDS/Normal (76.2%) was more than other syndromes (LKYDS/Normal, 17.0%; LGDHS/LDSDS, 31.3%; LDSDS/LKYDS, 22.2%). It suggests that these hub miRNAs of LGDHS/Normal and LDSDS/Normal networks might act more important roles than other TCM syndrome networks. 

The results implicate that these target genes were inhabited or degraded by upexpressing miRNAs and could deregulate the core cellular functions, such as immune responses and cell cycle in the molecular network. Actually, quantitative evaluation of module conservation in complex diseases using both gene expression and network connectivity as summation [25] provides a new avenue in understanding the molecular mechanism and distinguishing functional processes in disease progression [25, 26]. We speculate that such a hub-targeting mechanism may represent a more effective approach for TCM syndromes progression in CHB, which is from Excessive syndrome (LGDHS) to Excessive-deficient syndrome (LDSDS) and Deficient syndromes (LKYDS).

### 3.4. Networks Prioritize Target Genes and Pathways in TCM Syndromes Progression in CHB

In the biological network, one advantage is that network contains interaction information, as provided an intuitive way to explore gene functions in context using visualization approach. Such holistic approach has advantages for gene expression modules during both disease and its development for their regulation [27], and expression levels are the highest correlation across samples [28]. To understand these networks holistically, we conducted functional enrichment analysis for the target genes of the networks using DAVID analysis [21, 22]. Significant analysis was determined when *P* values were corrected for false discovery rate (FDR). Gene sets containing less than 5 genes overlapping were removed from the DAVID analysis. In our analysis, GO terms and pathways with an FDR-adjusted *P* value of less than 0.05 were retained. 

**Figure 4:**
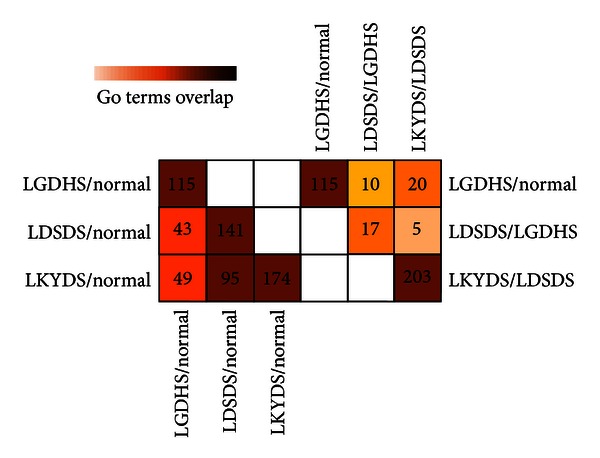
Comparison of network comprising GO terms. Numbers in the cell represent the number of overlapped GO terms in two networks. Colors were scaled according to the proportion of overlaps.

The representative GO terms of target genes in each TCM syndrome were shown in Figure 4. With the GO analysis, 43 GO terms were overlapping between LGDHS/Normal and LDSDS/Normal, and 49 GO terms are overlapping between LGDHS/Normal and LKYDS/Normal, while LDSDS/Normal and LKYDS/Normal have 95 GO terms in common. In addition, the overlap GO terms were also having greatly variety from Excessive syndrome (LGDHS) to Excessive-deficient syndrome (LDSDS) and then Deficient syndrome (LKYDS) progression. There were 10 GO terms overlapped between LGDHS/Normal and LGDHS/LDSDS, 5 terms were commonly between LGDHS/LDSDS and LDSDS/LKYDS, whereas LGDHS/Normal and LDSDS/LKYDS had 20 overlapped GO terms. The variety of this phenomenon was coincided to the relative network structure, suggesting that the TCM syndromes in CHB more likely had different molecular mechanisms.

As a DNA virus, HBV can be integrated into the host DNA and directed transforming hepatocytes [29]. This integration was induced a wide range of genetic alterations within the host genome, which may alter the expression of oncogenes, tumor suppressor genes, and microRNAs [30]. It has been also reported that the pathways related to cell death, DNA damage, DNA repair response, recombination, and signal transduction were predominant in HBV-infected liver [31, 32]. Here, their signaling pathway by each target genes was calculated using DAVID analysis too. 

Furthermore, the KEGG pathway (Tables 2 and 3) and BIOCART pathway (Tables 4 and 5) were analzed. As shown in Table 2, target genes were downregulated in TCM syndromes in CHB (miRNAs were upexpression levels). These genes of LGDHS/Normal were associated with the pathway of glioma and pathways in cancer, LDSDS/Normal main related to metabolism, RNA polymerase, and proteolysis, the LKYDS/Normal was related to cancer pathway, while cell cycle, p53 signaling pathway, and many cancer-related pathways main associated with LGDHS/LDSDS progression. Extraordinary, in Table 3, up-regulated target genes of Deficient syndrome much more related to cancer pathways. Compared with BIOCART pathway, the progression of Deficient syndrome was more like related to cell cycle (G1/S check point and G2/M checkpoint) and p53 signaling pathway. Many researches support that imbalance of G1/S and G2/M phases associated with dysfunction in hepatocarcinoma [33]. This result implicated that the Dxcessive-deficient and Deficient syndromes in CHB are more dangers for developed to cancer process. 

## 4. Conclusion 

In this study, through analyzing miRNA microarray date in CHB, we provided evidence that there are great difference among the LGDHS, LDSDS, and LKYDS based on classification of supervised learning algorithm and dynamic miRNA-target network. As the hierarchical cluster and SVM classifying, the Excessive syndrome (LGDHS) first cluster to Deficient syndrome (LKYDS), then as a parallel branch cluster to Excessive-deficient combination syndrome (LDSDS), the scores of each node are >80, implicated these TCM syndromes are distinctly variety on miRNA levels. The structure of dynamic miRNA-target network from Excessive to Deficient syndrome also demonstrated this remarkable difference. Analysis of the network node composing and network connection, denoted that the molecular mechanism has great varieties form Excessive to Deficient syndromes based on miRNAs expression levels among the 3 TCM syndrome networks. GO terms and pathway terms of prioritized target genes in networks revealed that the Excessive-deficient and Deficient syndromes in CHB are more dangers for developed to cancer process.

## Figures and Tables

**Table 1:** Differentiation of TCM syndromes in CHB patients.

Patient ID	Age	Gender	TCM syndromes	TCM syndrome types
LH1	32	M	LGDHS	Excessive
LH2	48	M	LGDHS	Excessive
LH3	46	F	LGDHS	Excessive
LH4	51	F	LDSDS	Excessive-deficient
LH5	59	M	LDSDS	Excessive-deficient
LH6	34	M	LDSDS	Excessive-deficient
LH7	55	F	LKYDS	Deficient
LH8	52	M	LKYDS	Deficient
LH9	50	F	LKYDS	Deficient

**Table 2:** KEGG terms distribution of downregulated genes (upexpression level of miRNAs) of TCM syndromes in CHB.

TCM syndromes	Kegg terms
LGDHS/normal	Glioma; pathways in cancer
LDSDS/normal	Pyrimidine metabolism; purine metabolism; RNA polymerase; ubiquitin-mediated proteolysis
LKYDS/normal	Pathways in cancer; TGF-beta signaling pathway; colorectal cancer
LGDHS/LDSDS	Cell cycle; chronic myeloid leukemia; p53 signaling pathway; pathways in cancer; pancreatic cancer; colorectal cancer; bladder cancer; glioma; prostate cancer; small cell lung cancer; melanoma; nonsmall cell lung cancer; renal cell carcinoma; TGF-beta signaling pathway; ErbB signaling pathway; insulin signaling pathway
LDSDS/LKYDS	Cell cycle; colorectal cancer

**Table 3:** KEGG terms distribution of upregulated genes (downexpression level of miRNAs) in CHB syndromes.

Syndromes	Kegg term
LGDHS/normal	Ribosome; prostate cancer; aminoacyl-tRNA biosynthesis; small cell lung cancer; insulin signaling pathway; glioma; pathways in cancer
LDSDS/normal	Pathways in cancer; small cell lung cancer
LKYDS/normal	Pathways in cancer; cell cycle; prostate cancer; colorectal cancer; TGF-beta signaling pathway; p53 signaling pathway
LGDHS/LDSDS	Prostate cancer; neurotrophin signaling pathway
LDSDS/LKYDS	Pathways in cancer; bladder cancer; steroid biosynthesis; pancreatic cancer; chronic myeloid leukemia; nonsmall cell lung cancer; colorectal cancer; adipocytokine signaling pathway; insulin signaling pathway; cell cycle; prion diseases

**Table 4:** BIOCART terms distribution of downregulated genes (upexpression level of miRNAs) of TCM syndromes in CHB.

Syndromes	Biocart term	Genes
LGDHS/normal	Hypoxia-inducible factor in the cardiovascular system	EP300, VHL, CREB1, VEGFA, ASPH
LDSDS/normal	Control of gene expression by vitamin D receptor	COPS2, BAZ1B, SMARCC1, SMARCD1, CHAF1A, SMARCA4
LKYDS/normal	Melanocyte development and pigmentation pathway	CREB1, BCL2, MITF, KITLG, KIT
LGDHS/LDSDS	Cell cycle: G1/S checkpoint	E2F1, SMAD4, SKP2, CDK6, RB1, CDK4, ATM, CDC25A, CCND1, CDKN1A, CDKN1B, DHFR, CDKN2A, HDAC1, CDKN2B
	p53 signaling pathway	E2F1, CDKN1A, CCND1, BCL2, PCNA, APAF1, RB1, CDK4, TIMP3, GADD45A, ATM
	Cyclin, cell cycle regulation	E2F1, CCNB1, CDKN1A, CCND1, CDKN1B, CDKN2A, CDKN2B, CDK6, RB1, CDK4, CDC25A
	Cell cycle: G2/M checkpoint	CCNB1, CDKN1A, YWHAH, EP300, CHEK1, MYT1, GADD45A, ATM, BRCA1, WEE1
LDSDS/LKYDS	Role of BRCA1, BRCA2, and ATR in cancer susceptibility	RAD1, NBN, BRCA2, BRCA1, RAD51
	Cyclin, cell cycle regulation	CCNE1, CDKN2A, CDKN2D, RB1, CDC25A

**Table 5:** BIOCART terms distribution of upregulated genes (downexpression level of miRNAs) of TCM syndromes in CHB.

Category	Term	Genes
LGDHS/normal	Prion pathway	RPSA, BCL2, LAMC1, DNAJB1, HSPA5, LAMB1, PRNP
LDSDS/normal	Signaling pathway from G-protein families	RPS6KA3, GNAQ, MAP2K1, GNB1, JUN, PPP3CA
LKYDS/normal	ALK in cardiac myocytes	TGFBR1, SMAD5, TGFBR2, SMAD4, BMPR2, BMP7, ATF2, BMPR1A, ACVR1, APC
	Melanocyte development and pigmentation pathway	CREB1, BCL2, MITF, KITLG, KIT
LGDHS/LDSDS	No	No
LDSDS/LKYDS	Cell cycle: G1/S check point	E2F1, SMAD4, SMAD3, CDK6, RB1, CDK4, ATM, CDC25A, CCND1, CDKN1A, CDKN1B, DHFR, CDKN2A, HDAC1, CDKN2B, CCNA1
	p53 signaling pathway	E2F1, CDKN1A, CCND1, BCL2, BAX, PCNA, APAF1, RB1, CDK4, TIMP3, ATM
